# Optimization of the co-digestion of sewage sludge, maize straw and cow manure: microbial responses and effect of fractional organic characteristics

**DOI:** 10.1038/s41598-019-38829-8

**Published:** 2019-02-20

**Authors:** Liangliang Wei, Kena Qin, Jing Ding, Mao Xue, Chaoyong Yang, Junqiu Jiang, Qingliang Zhao

**Affiliations:** 1 State Key Laboratory of Urban Water Resources and Environment (SKLUWRE), School of Environment, Harbin Institute of Technology, Harbin, 150090 China; 2 Tianjin Municipal Engineering Design & Research Institute, Tianjin, 300051 China

## Abstract

The aim of this study was to evaluate the efficiency and optimization of co-digestion using sewage sludge (SS), maize straw (MS) and cow manure (CM) as feeds, and the effects of the mixing ratio and C/N ratio of the substrates were analyzed in detail. Among the three substrates tested, CM/MS exhibited better digestion than CM/SS and SS/MS in terms of all measures, including total daily biogas and net methane volume production, due to the hydrophilic characteristics and high level of biodegradability of CM, as well as its higher C/N ratio. The average biogas production was 613.8 mL/g VS for the co-digestion of CM/MS at a feed concentration of 15 g VS/L and using a 1:1 mixing ratio (C/N ratio of 28.3). The co-digestion of SS/CM/MS performed better than the individual digestion of the components because of the balanced C/N ratios and supply of carbon. The optimum conditions for maximizing methane potential were an SS:CM:MS ratio of 30:35:35 and a bulk VS concentration of 15.0 g VS/L, which led to a maximum methane production of 8047.31 mL (C/N ratio of 12.7). The high-throughput sequencing analysis showed clear differences in microbial communities during the entire co-digestion process.

## Introduction

The continuous development of the animal husbandry, wastewater treatment, and agricultural industries in China has resulted in increasing discharges of cow dung, sewage sludge, and maize straw into the environment^1^. The irresponsible disposal of these solid wastes has caused serious toxic effects on aquatic ecosystems, especially in northern China; thus, a proper disposal method is required to meet ever-increasing legislative standards^2^. Among all of the disposal processes, anaerobic digestion has attracted much attention in recent years due to its efficient reduction of organic matter and energy extraction (CH_4_ and H_2_)^3^. In comparison to traditional aerobic approaches, the behavior of anaerobic digesters is always restricted by nutrient sources, operational temperature, hydraulic retention time (HRT), and the presence of inhibitors^4,5^.

To improve the performance of anaerobic digesters, the co-digestion of different organic wastes, such as activated sludge, kitchen waste, vegetables, livestock, straw and poultry dung, has attracted much attention in recent years^6,7^. Gelegenis *et al*. recently observed that the co-digestion using diluted poultry manure and whey as substrates (V/V = 65:35) led to a 40% increase in CH_4_ production^8^. Alvarez and Lidén found that the co-digestion of sheep, llama and cow manure resulted in methane production (in m^3^ CH_4_/kg VS) of 0.14, 0.10 and 0.09 for a 50–50% mix of llama and sheep, cow and sheep, and llama and cow manure, respectively, under an organic loading level of 1200 g VS m^3^/d^9^. Wu *et al*. reported optimal digestion when pig manure and cereal straws were mixed under a C/N ratio of 20^10^, while the optimal C/N mixing ratio was 33 for cassava pulp and pig manure co-digestion^11^. To further enhance biogas production, the synergetic effects of the proportions of mixed substrates on the performance of batch digesters were recently evaluated using statistical techniques^12^.

Traditionally, the organics removal rate and biogas productivity are usually considered the two important parameters for judging the performance of digesters^13^. However, the traditional COD measurement is not as useful because of its time-lapse response and significant daily variation due to the colloidal/polymer characteristics of the organic feeds of sewage sludge, kitchen waste, livestock, etc.^14,15^. Thus, the parameters of soluble chemical oxygen demand (SCOD), extracellular polymeric substances (EPS), and extracellular biological organic matter (EBOM) have recently been applied as novel approaches for interpreting the removal of organics during sludge digestion^14^. Recently, XAD resin fractionation has also been applied to study the removal trend of organics during anaerobic digestion; accordingly, detailed information on the hydrophobic/hydrophilic polarity distribution of organic waste before and after digestion, as well as which part of the organic material was efficiently converted, has been clearly observed^16^. Early work by our group found that a hydrolyzation rate ratio of less than 1.0 for the hydrophobic acid (HPO-A) and hydrophilic fraction (H_HPO-A_-to-H_HPI_) in sludge EPS could be used as a proxy for the poor hydrolyzation state in digesters^17^. Thus far, most research has focused on optimizing the operational parameters of the co-digesters, whereas it has rarely focused on the transformation of fractional organics during anaerobic co-digestion or on the microbial responses.

Here, lab-scale anaerobic digesters were operated using different substrates and C/N ratios via the gradual changes in the sewage sludge (SS), cow manure (CM) and maize straw (MS) mixing ratios. The first objective of the present study was to assess the methane production rate and organic removal rate of the co-digesters under different operation parameters. Then, the fractional characteristics of NH_4_OH extractable organics of the feed before and after digestion were assessed. Finally, the sequenced genome of the biomass within the reactors was periodically analyzed to identify the variation in microbial communities during the entire co-digestion process.

## Results

### Chemical composition of the feeds of anaerobic digesters

**Figure 1:**
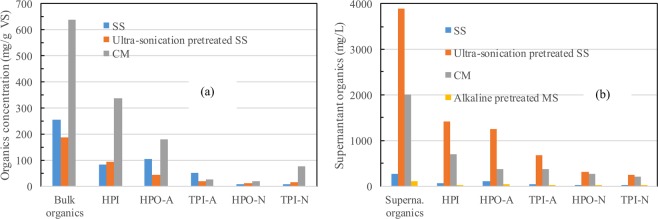
Fractional DOC distribution of residual organics (**a**) and supernatant organics (**b**) for the feeds of SS, ultrasonicated SS, CM and alkaline-pretreated MS.

As shown in Fig. 1, the majority of the organics within the sewage sludge (SS) existed as polymeric EPS (256 mg DOC/g VS), and only a small portion existed in the supernatant sludge (267.2 mg/L). Specifically, HPO-A (41.2%) and HPI (32.7%) were the predominant fractions of the SS-related EPS, whereas that of the other three fractions decreased in the trend of TPI-A (19.3%) > TPI-N (3.6%) > HPO-N (3.3%). In contrast, the fractional organics of the supernatant sludge were distributed as HPO-A (37.5%) > HPI (27.3%) > TPI-A (14.9%) > HPO-N (11.8%) > TPI-N (8.5%). To disintegrate the polymeric EPS, the sludge organics were ultrasonicated, and the experimental results demonstrated that the SCOD of the supernatant sludge increased from 267.2 mg/L to 3890.3 mg/L under an ultrasonic density of 1.5 W/mL (pH 7), accompanied by an EPS concentration that decreased from 256 to 186 mg DOC/g VS. Meanwhile, the HPO/HPI ratio of the sludge EPS decreased from 1.36 to 0.60 when the sonication process progressed; accordingly, the percentage of the hydrophilic organics of the supernatant sludge increased from 27.3% to 36.5% instead.

CM exhibited a relatively higher organic content of 636.9 mg/g VS, in which the hydrophilic fraction contributed as much as 52.9% of the bulk DOC, followed by HPO-A (28.4%). Similarly, HPI and HPO-A were also the main components of the supernatant CM, accounting for as much as 34.7% and 18.6% of the bulk organics. Compared to that of SS, the higher content of organics within CM (especially hydrophilic CM) theoretically implied a higher methane yielding potential. To efficiently enhance the destruction of the polymer structure, MS was pretreated with 6% NaOH, and the experimental results showed that the contents of lignin and hemicellulose declined from 10.38% and 32.5% to 6.42% and 16.6%, respectively, in the residual solids. The supernatant organics of the NaOH-pretreated MS feed exhibited a hydrophobic characteristic (43.3% for HPO-A and 17.0% for HPO-N) due to the higher lignin content^18^. Undoubtedly, the transfer of the alkaline soluble organics from the solid to the supernatant after NaOH pretreatment benefited the anaerobic digestion of MS.

Meanwhile, noticeable differences were observed in the biodegradability of the feeds of CM, alkaline-pretreated MS, and SS (raw SS and ultrasonicated SS); the higher the HPO/HPI ratio was, the lower the corresponding biodegradability was (Table 1). The above results were quite similar to our previous observation that a higher ratio of hydrophobic/hydrophilic fractions in the sludge could be an indicator for the poor biodegradability of organic feeds^17^. In general, MS exhibited hydrophobic polarity and correspondingly refractory characteristics (BDOC/DOC = 33.5%). In comparison, the BDOC/DOC ratio of the other three feeds exhibited a decreasing trend of CM (60.7%) > ultrasonicated SS (54.2%) > SS (37.3%) > MS (33.5%).

**Table 1:** The HPO/HPI distribution and biodegradability of feeds of SS, CM and MS.

	Ratio of HPO-/HPI	Residual organics	Biodegradation rate (%)
	Supernatant organics		Supernatant organics
SS	1.80	1.36	37.3
Ultrasonicated SS	1.10	0.60	54.2
CM	0.93	0.59	63.2
Alkaline pretreated MS	2.59		33.5

### Effect of substrate mixing ratios and C/N ratios on co-digestion performance of CM, MS and SS

**Figure 2:**
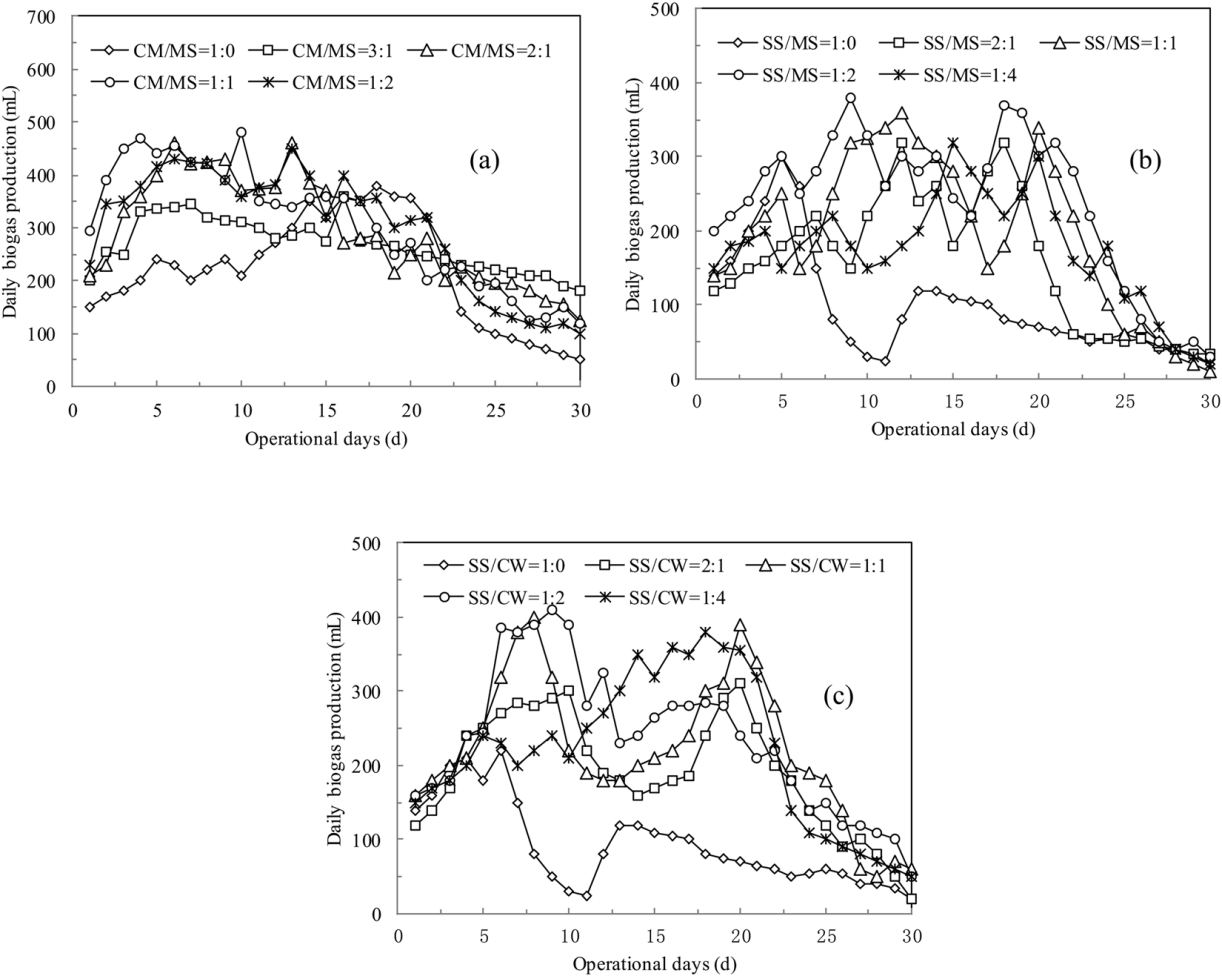
Daily biogas production rate of the co-digestion of CM/MS (**a**), SS/MS (**b**) and SS/CW (**c**) using different feed ratios.

The performance of the digesters varied widely depending on the feed ratios of CM/MS, SS/MS and SS/CW (Fig. 2). Taking the co-digestion of CM/MS as an example, the removal trends of VS were 55.4%, 58.2%, 63.2%, 58.6% at CM/MS ratios of 3:1, 2:1, 1:1 and 1:2, respectively, which are much higher than the mono-digestion of CM (45.8%). Meanwhile, the co-digestion of CM/MS shortened the lag phase, and only 3, 6 and 7 days were needed to reach the maximum methane production using 1:1, 2:1 and 3:1 CM/MS mixing ratios (17 days were needed for CM mono-digestion). The average biogas volumetric production was 613.8 mL/g VS for the 1:1 CM/MS (C/N = 28.31) co-digestion, 1.40 times higher than that using CM as a mono-substrate (C/N = 21.28). However, the further adjustment of CM/MS to 1:2 decreased the bulk methane production (603.46 mL/g VS), which was related to the high content of nonbiodegradable lignin in MS^19^. In general, the MS additive balanced the C/N ratio and improved the performance of the co-anaerobic digesters.

The average biogas productivity of the co-digestion of SS/MS was lower than that of CM/MS, despite a comparable VS removal rate, which was related to the relatively lower biogas production potential of 198.3 mL/g VS of the sewage sludge. As shown in Fig. 2b, as much as 55.8%, 57.4%, 58.9%, and 58.1% of the VS was efficiently removed using an SS/MS ratio of 2:1, 1:1, 1:2 and 1:4, respectively, whereas the corresponding biogas production rates were 336.1, 395.2, 472.3 and 352.3 mL/g VS, respectively. Two significant plateaus of methane productivity were observed for the co-digestion of SS/MS independent of the feed mixing ratio; as they only appeared on the 5^th^ day of the sewage sludge mono-digestion, they clearly demonstrated that the addition of MS to the digesters prolonged the duration of biogas production.

The removal trend of organics and the biogas productivity of the co-digestion of SS/CM were similar to that of SS/MS (Fig. 2c), and they both exhibited two plateaus of methane production during the 30-day operation. It should be noted that the mono-digestion of CM and SS required 18 days and 6 days, respectively, to reach the maximum CH_4_ production; thus, the two plateaus of methane production observed during the co-digestion of SS/CM were related to the varied hydrolyzation potential of the sewage sludge and cow manure. The average COD removal rate of the SS/CM was 47.2%, 43.2%, 57.1%, and 53.1% during steady-state digestion using mixing ratios of 2:1, 1:1, 1:2, and 1:4, respectively, whereas a lower methane production was observed than that of CW/MS (352.3–470.3 mL/g VS *versus* 534.8–613.8 mL/g VS). In general, the optimal mixing ratio was 1:2 for CM/SS co-digestion, and the corresponding average methane production was 7% higher than that of CM as a mono-feed and 137% higher than that of SS.

### Performance of co-digesters and factorial experimental design

As shown in Table 2, the co-digestion of SS, CM and MS significantly enhanced CH_4_ productivity compared to sewage sludge digestion. In summary, anaerobic digestion using sewage sludge as a mono-substrate (D14 in Table 2) was unstable, and a total of 2975 mL biogas was collected after 30 days of operation (partially related to its lower C/N ratio of 5.32), which was much lower than the data obtained from the co-digestion tests of D1-D12. Undoubtedly, the addition of CM or MS to the digesters for sewage sludge degradation efficiently enhanced the conversion of organics to CH_4_. The possible mechanisms could be summarized as follows: (1) the sufficient hydrophilic organics supplied from CW; (2) the buffering capacity originating from the CM additive; and (3) the rebalancing of C/N and a decrease in ammonia toxicity^20^. The factorial experimental results demonstrate that the co-digestion of CM/MS led to a higher biogas productivity compared to that of SS/MS and SS/CM, yielding a maximum CH_4_ production rate (5738 mL) under the condition of a 1:1 CM/MS mixing ratio and a 15 g VS/L substrate concentration (D3 test, C/N = 28.3 shown in Table 2), which was consistent with the previous finding of the optimal ratio of C/N = 27.2:1 for the co-digestion of cow and chicken manure^21^.

**Table 2:** Performance of digesters under the mono- and co-digestion of SS, CM and MS.

Item Unit	Digestion Type	Bulk Concen. (g VS/L)	CM/MS	SS/MS	SS/CM	C/N	Total Biogas (mL)	Methane yield (mL/g VS)	Total methane (mL)	Ratio of Methane (%)
D1	Co-digestion	15.0	3:1			23.99	8022	534.8	4108	51.21
D2	Co-digestion	15.0	2:1			25.21	8817	587.8	5058	57.37
D3	Co-digestion	15.0	1:1			28.31	9207	613.8	5738	62.32
D4	Co-digestion	15.0	1:2			32.84	9052	603.46	5310	58.66
D5	Co-digestion	15.0		2:1		7.33	5040	336.0	2458	48.8
D6	Co-digestion	15.0		1:1		9.19	5925	395.0	3124	52.7
D7	Co-digestion	15.0		1:2		12.50	7085	472.3	4288	60.5
D8	Co-digestion	15.0		1:4		17.82	5285	352.3	2733	51.7
D9	Co-digestion	15.0			2:1	7.46	5540	369.3	2682	48.4
D10	Co-digestion	15.0			1:1	9.10	6580	438.7	3562	54.1
D11	Co-digestion	15.0			1:2	11.43	7055	470.3	4140	58.7
D12	Co-digestion	15.0			1:4	14.15	5285	352.3	2733	51.7
D13	Mono-digestion	15.0	1:0			21.28	6585	439	2769	42.05
D14	Mono-digestion	15.0		1:0		5.32	2975	198.33	1217	40.9

### Parameter optimization of the co-digestion of SS/CM/MS

According to the above section, the optimal mixing ratio of 1:1 was selected for the co-digestion of CW/MS; thus, the interaction effects of the SS additive on the co-digestion behavior of SS/CM/MS were studied under the condition of CM/MS = 1:1. As shown in the Supplementary Table S1, increasing the SS additive from 20% to 30% enhanced the methane productivity and simultaneously improved the removal of organics, whereas further increasing the mixing ratio to 50% negatively affected CH_4_ production. For example, the bulk methane production yields were 7425, 8052, and 7658 mL for the SS/CM/MS co-digestion (mixing ratio 30:35:35) using substrate concentrations of 10, 15 and 20 g VS/L, respectively, after 30 days of steady-state operation, which were higher than the corresponding data obtained from the 50% sewage sludge additive tests (6958, 7235 and 7010 mL, respectively). In general, the efficiency of anaerobic digestion was always restricted by the imbalance in carbon sources^17^; thus, a higher percentage of the sludge additives within the co-digestion system decreased CH_4_ productivity and subsequently restricted the performance of the digesters. In general, maintaining a mixing ratio of 30% for SS/(CM + MS) and a 15.0 g VS/L feed concentration yielded the maximum methane productivity of 8052 mL.

**Figure 3:**
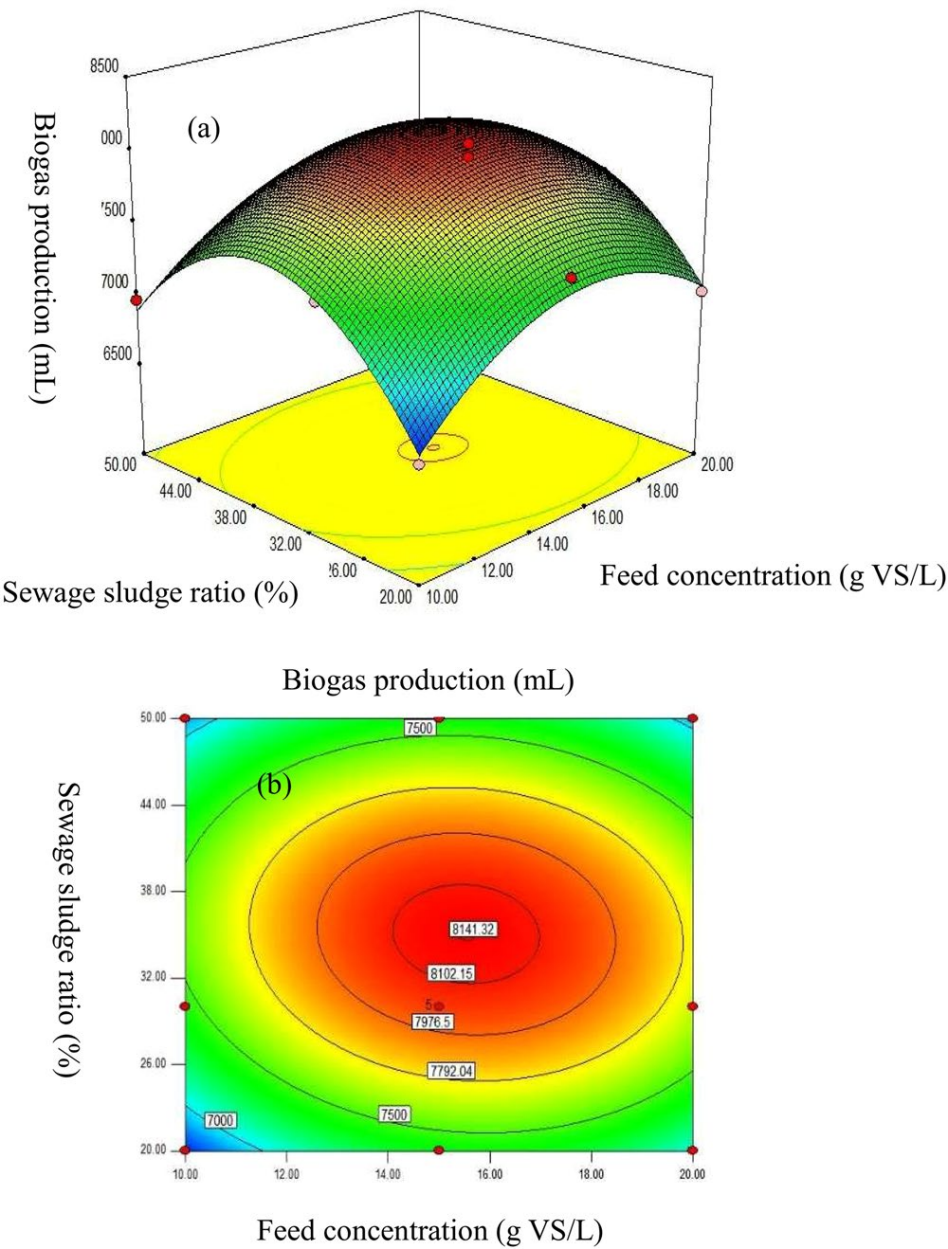
Response surface and contour plots for methane potential depending on variations of SS additive and feed concentrations.

To further evaluate the synergetic effects of the proportions of mixed substrates on the performance of batch digesters, the parameter optimization of the co-digestion of SS/MS/CM was investigated using response surface methodology (RSM), and the results are presented in Fig. 3. A second-order polynomial equation for methane yielding potential was obtained:

1 $$Y=8136.51+103.09\times A+13.67\times B-81.70\times A\times B-481.09\times {A}^{2}-761.78\times {B}^{2}$$

where *Y* is the predicted biogas production rate (mL), *A* refers to the bulk concentration of feeds (g VS/L), and *B* presents the ratio of SS/(CM + MS) (%). A relatively high regression rate of R^2^ = 0.9787 indicated that the model fit the majority of data obtained the co-digestion tests well. According to the response surface optimization, the highest methane production rate could reach 8141.32 mL at a 30.06% SS/(CM + MS) ratio and a 15.54 g VS/L feed concentration.

### Transformation of the organic fractions of SS, CM and MS during co-digestion

**Figure 4:**
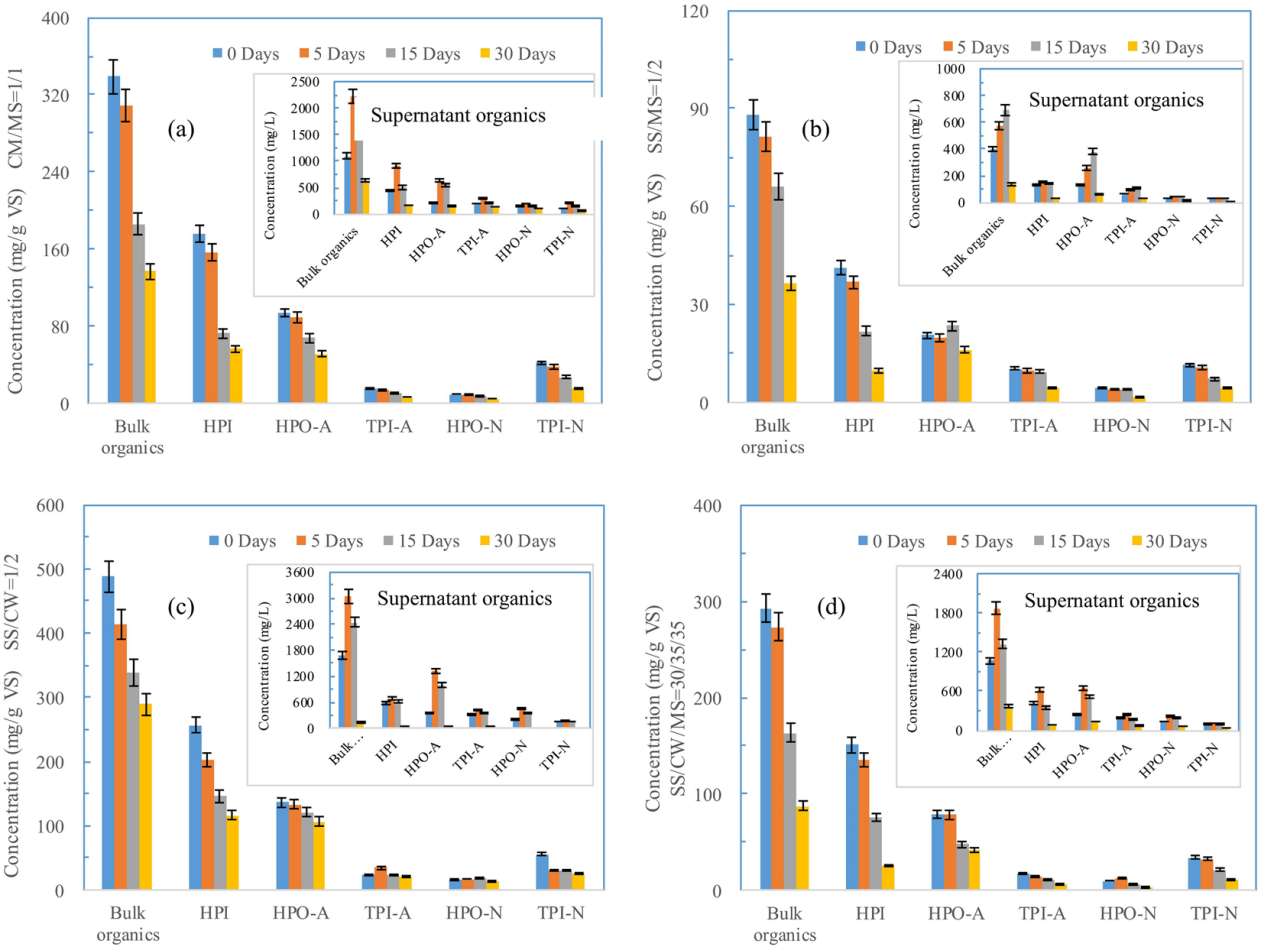
Removal trends of NH_4_OH-extractable organics and their fractions during the co-digestion of CW, SS and MS (**a**) CM/MS 1:1; (**b**) SS/MS 1:2; (**c**) SS/CM 1:2; (**d**) SS/CM/MS 30:35:35.

The removal trend of the NH_4_OH extractable organics in the feeds varied widely depending on both the feed chemical characteristics and their mixing ratios (Fig. 4). In summary, as much as 8.8%, 45.0% and 59.6% of the organics in the digester using CW and MS as feed (CW/MS = 1:1) was efficiently hydrolyzed after 5 days, 15 days and 30 days of co-digestion, respectively. Specifically, hydrophilic feeds were preferentially hydrolyzed compared to the hydrophobic components under each condition, as indicated by the relatively higher removal rates of 10.7%, 58.6% and 67.8% obtained from 5, 15 and 30 days of digestion, respectively. In general, the hydrolyzed organics, especially the NH_4_OH-extractable hydrophobic organics, led to a significant DOC concentration increase of the supernatant organics during the initial 15 days of co-digestion (210.9% and 169.1% for HPO-A after 5 and 10 days of digestion compared to 107.6% and 14.4% for HPI, respectively). In addition, as much as 86.5% of the HPI of CW + MS (easily biodegradable) could be efficiently hydrolyzed and biodegraded within the initial 15 days of digestion, much higher than that of HPO-A (61.8%), which might be the main reason why a significant decline in methane productivity was observed during the subsequent 16–30 days of co-digestion.

The concentration of the NH_4_OH-extractable organics of the SS/MS (1:2 mix ratio) feed did not change significantly during the initial 15 days of digestion (Fig. 4b), differing widely from those of SS/CW and CW/MS. Specifically, the hydrophobic organics of the supernatant feed increased significantly after 15 days of co-digestion, demonstrating that it needed a long time for the complete destruction of the maize straw-related lignin polymers during the entire co-digestion process. In addition, a relatively lower concentration increase in supernatant organics was obtained from the 5- and 15-day operated digesters (44.5% and 73.7%, respectively) implying that the hydrolyzation process was the rate-limiting factor of the co-digestion process of SS/MS.

As shown in Fig. 4(c), the removal trends of the organic fractions of SS/CW (mixing ratio of 1:2) were similar to those of CW/MS during the 30-day operation. In general, the relatively lower removal rate of 40.7% of the feed organics was observed during the 30-day digestion of CW/MS due to the relatively lower hydrolyzation of the HPO-A-related organics. It should be noted that SS/CW exhibited hydrophilic characteristics and a relatively high concentration of supernatant organics, which might be the main reason why the hydrolyzed organics of SS/CM could be easily utilized for CH_4_ generation. For example, the hydrophilic components of the supernatant portion of SS/CW declined by 94.9% when the 30-day co-digestion process progressed and by 83.9% for HPO-A. Thus, we can conclude that the methanogenesis steps were not the rate-limiting factor for the SS/CW co-digestion, and the relatively lower removal of organics might be ascribed to the inefficient hydrolyzation caused by the unbalanced C/N ratio and acid accumulation.

In general, the co-digestion of SS/CW/MS under an optimal mixing ratio of 30:35:35 guaranteed a relatively higher organic removal rate regardless of the chemical characteristics of the organic fractions, benefiting from C/N balancing and the sufficient supply of hydrophilic nutrients. As shown in Fig. 4d, both the hydrophobic and hydrophilic organics within the feeds of SS/CW/MS could be efficiently hydrolyzed (evidenced by the sharp decline in the COD of the solid organics) after 15 days of steady-state operation, and the insignificant accumulation of supernatant organics implied that those hydrolyzed organics could also be efficiently converted to CH_4_. In general, as much as 70.1% of the feed organics were efficiently biodegraded after 30 days of co-digestion, whereas those for HPI and HPO-A were 83.1% and 47.8%, respectively; this was the highest among all 4 selected co-digesters listed in Fig. 4.

### Microbial responses during the optimized operation of co-digestion

The taxonomic diversity of the bacterial and archaeal methanogens of the co-digesters using SS/MS, SS/CM, MS/CM and MS/SS/CM as feed as indicated by mcrA and archaeal 16S rRNA genes was analyzed with 454 pyrosequencing.

#### Bacterial community composition

After 15 days of steady-state operation, a total of 20137, 23045 and 22155 bacterial OTUs were detected within the digesters using SS/MS, SS/CM and MS/CM as feeds (maximum CH_4_ production), and those bacterial OTUs further increased to 30211, 31612 and 32314 when the operation period was extended to 30 days. The above results clearly demonstrated that a diverse community of biofilms existed within the steady-state-operated co-digesters. The bacterial OTU number of the digester using SS/CM/MS (30:35:35) was 30412 after 30 days of cultivation, and the noteworthy archaeal methanogen growth might be the main reason for the significant CH_4_ conversion.

**Figure 5:**
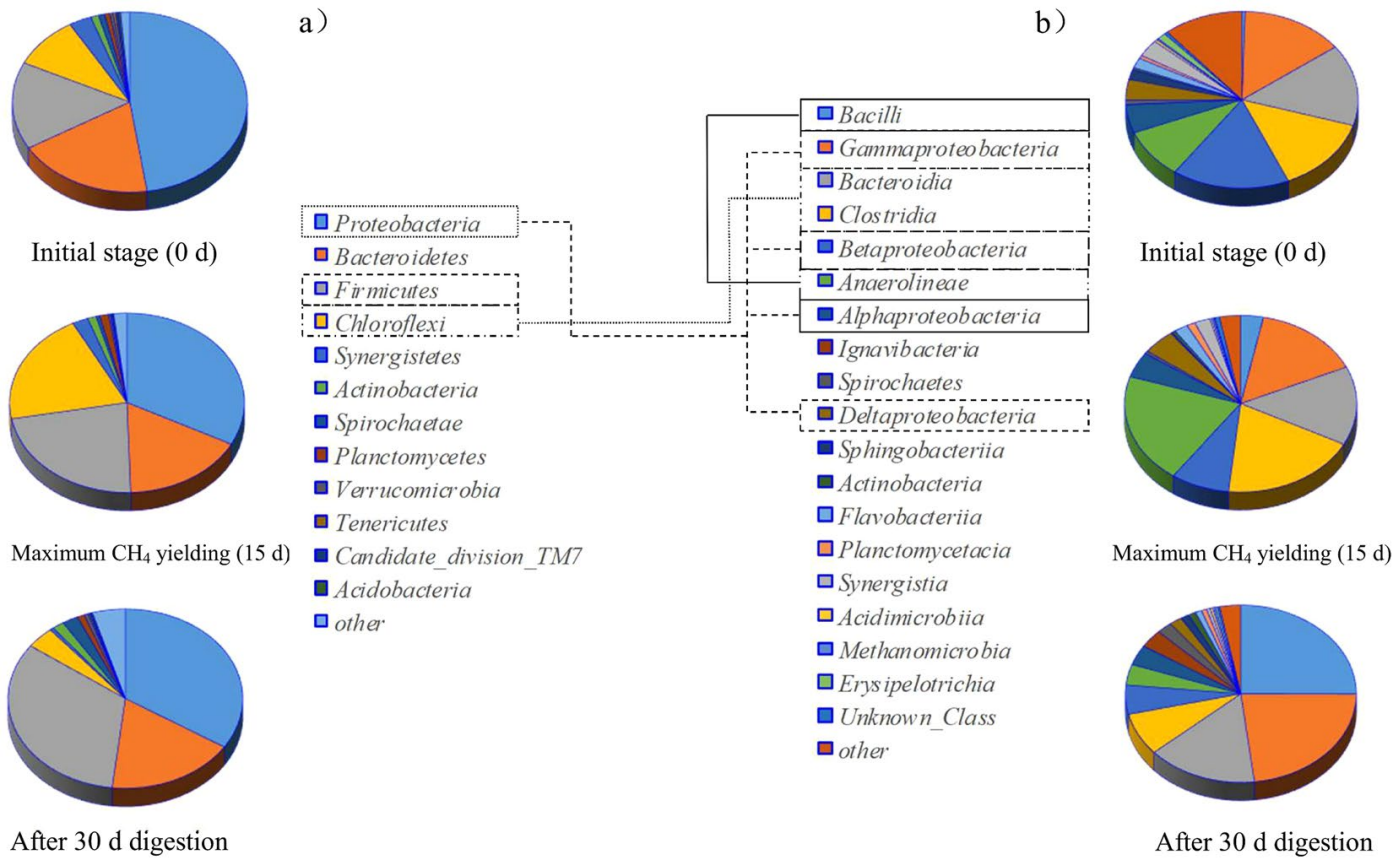
Bacterial community composition (**a**) phylum, and (**b**) class of the co-digester at the initial stage, at the maximum CH_4_ yielding condition and after 30 days of digestion.

Because the distribution of the bacterial community of the CM/MS, SS/CW, and SS/MS co-digestion was similar to that of CM/MS/SS, the species and distribution of the biomass of the digester using CM/MS/SS as feeds were selected and discussed here. Figure 5a shows the relative abundance of the taxa comprising at least 1% in at least one sample. Specifically, Protecbacteria, Firmicutes, Chloroflexi and Bacteroidetes were the four dominant microorganisms (genus level) within the inoculated feed, and the percentage distributions of those four species differed widely and decreased in the trend of Protecbacteria (47.64%) > Bacteroidetes (18.85%) > Firmicutes (15.65%) > Chloroflexi (9.34%). A slight percentage decline of 91.48% to 88.93% was observed for the above mentioned four species after 15 days of steady-state co-digestion, which further increased to 92.36% when the 30-day operation progressed. In general, the percentage distribution of the predominant species changed significantly during the initial 15 days of co-digestion, which is indicated by the significant percentage increase of Firmicutes and Chloroflexi (from 15.65% to 22.6% and 9.34% to 20.27%, respectively). Ziganshin *et al*. and Regueiro *et al*. found that Firmicutes and Chloroflexi efficiently hydrolyze refractory cellulose and carbohydrates into soluble acidic during anaerobic digestion^22,23^, which might be the main reason for the significant decomposition of those polymer organics during the first 15 days of co-digestion. In contrast, Protecbacteria, which played a major role in the biodegradation of the sludge proteins, decreased from 47.64% to 32.65% instead, which was related to the C/N increase caused by the addition of MS and CM. In comparison, the occurrence of the well-known, extremely resistant microorganisms of Bacteroidetes, which can degrade complex organisms^24,25^, did not change significantly.

#### Archaeal community composition

**Figure 6:**
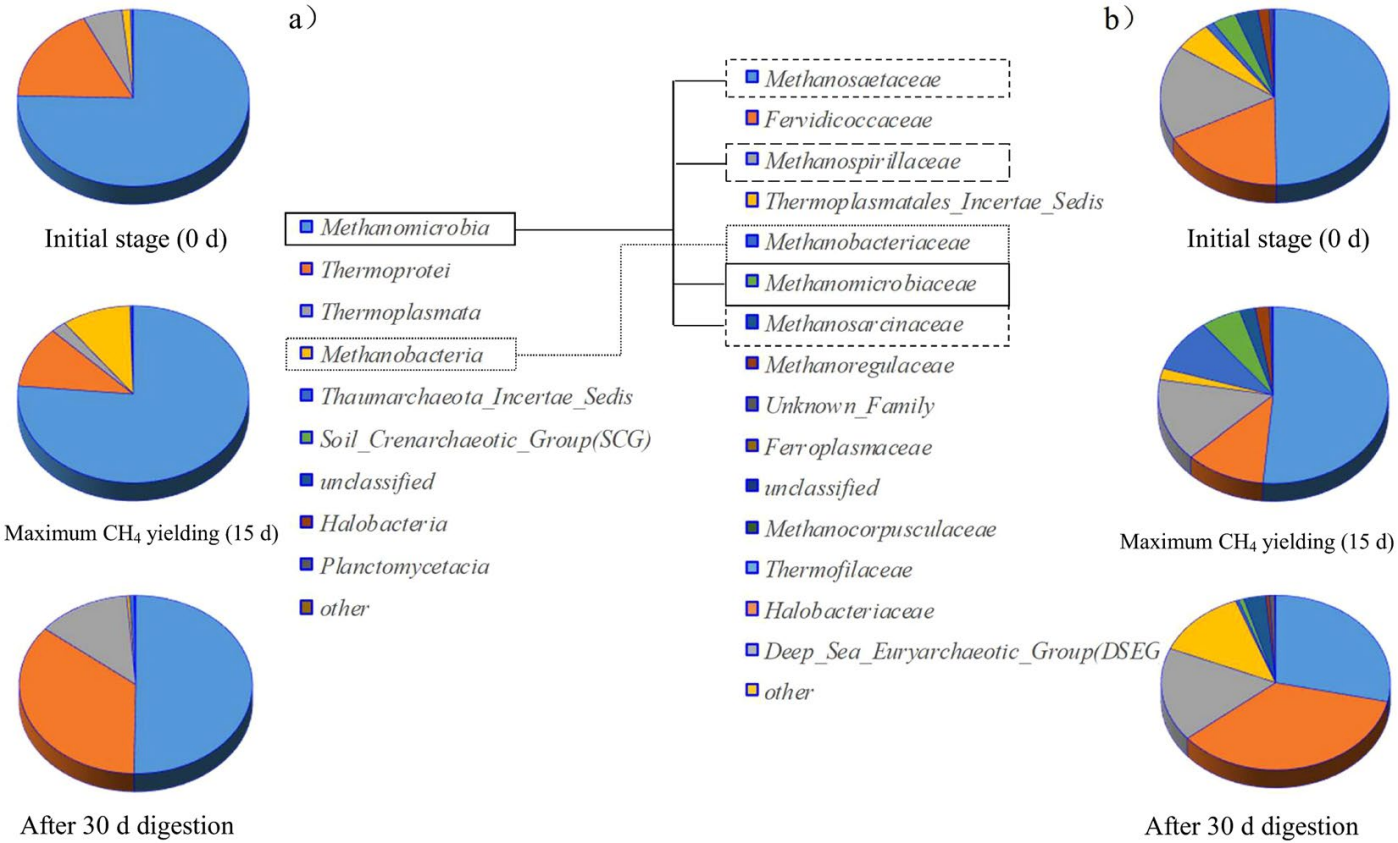
Archaeal community composition (**a**) phylum, and (**b**) class of the co-digester at the initial stage, at the maximum CH_4_ yielding condition and after 30 days of digestion.

In total, 26508 archaeal clones were collected from the inoculated digested sludge and the OTUs were 30316 and 75432 for the 15- and 30-day operated digesters, respectively. As shown in Fig. 6, *Methanosaetaceae*, producing CH_4_ utilizing acetic acid, H_2_ and CO_2_, was the dominant species in the anaerobic digester. The percent abundance of aceticlastic *Methanosaetaceae* increased from 49.75% to 51.42% after 15 days of digestion (reached maximum methane productivity). According to the recently published work of Karakashev *et al*.^26^, the C/N balancing of anaerobic digesters generated from the MS/CM additive, as well as low-ammonia conditions, undoubtedly benefited the performance of *Methanosaetaceae*. In comparison, the abundance of hydrogenotrophic methanogens of *Methanobacteria* and *Methanomicrobiales* increased from 1.21% and 3.41% to 9.4% and 5.7%, respectively, demonstrating that hydrogenotrophic methanogenesis also played a great role during the conversion of those hydrolyzed organics into CH_4_. In addition, the percentage of *Methanosarcinaceae* (the dominant aceticlastic groups) decreased from 3.24% to 2.22%, and those of the other species did not change significantly.

## Discussion

The cultivation of the feed of CM/MS (1:1) yielded the highest methane volume (5738 mL) among the three co-digestion processes of CM/MS, SS/MS and SS/CM using a 15 g VS/L feed concentration. The corresponding C/N ratio was approximately 28.3, similar to the previous findings that an optimal C/N ratio of 27.2:1 was observed for the cow and chicken manure co-digestion^21^. In general, the feed of CM exhibited a higher organic content (636.9 mg/g VS) and could be easily hydrolyzed, whereas the feed of MS exhibited a higher C/N ratio of 53.3 (despite hydrophobic characteristics). Thus, the balancing of the C/N ratio after adding MS to the feed was the main reason for the higher CH_4_ productivity of CM/MS co-digestion. From the above data, we can conclude that the optimization of the C/N ratio was an effective approach for enhancing the efficiency of the co-digestion system.

The addition of biodegradable organics, especially hydrophilic components, also improved the methane production for anaerobic co-digestion. As shown in Table 1, the biodegradability of the feeds decreased as follows: CM (60.7%) > ultrasonicated SS (54.2%) > SS (37.3%) > alkaline-pretreated MS (33.5%). Specifically, the readily biodegradable CM exhibited a relatively higher content of hydrophilic organics regardless of the characteristics of the supernatant components and residual organics (which were 0.93 and 0.59 for HPO and HPI, respectively), in which the fraction of the HPI accounted for as much as 52.9% of the bulk organics of the NH_4_OH-extractable organics. Traditionally, the majority of hydrophilic organics consist of polar compounds with a low molecular weight^17,27^; thus, the lower the ratio of HPI/HPO in the organic feeds, the poorer the biodegradability during anaerobic digestion, which might be the main reason for the highest methane productivity being observed from the mono-digestion using CM as feed. In contrast, the higher HPO/HPI ratio of the SS-related organics (1.80 in supernatant and 1.36 in residual organics) and the refractory characteristics (BDOC/DOC = 33.5%) of MS might be the main reason for the lower CH_4_ productivity. From the above, we can conclude that the refractory characteristics of the hydrophobic organics in the feeds restricted the entire methanogenesis step of the co-digestion process.

In addition, the significant destruction/solubilization of the polymer hydrophobic components of the SS and MS feeds, as observed from the ultrasonication and alkaline pretreatment of SS and MS, would enhance the bulk CH_4_ productivity. Moreover, the decrease in the HPO/HPI ratios of the supernatant organics of the feeds that was observed after pretreatment demonstrated that the ultrasonication and alkaline pretreatment promoted the conversion of hydrophobic SS and MS to soluble hydrophilic supernatant sludge, which was meaningful for the entire anaerobic co-digestion.

The microbial community structure distribution and variation partially revealed the performance of the co-digestion processes and the removal characteristics of the organics. In general, the occurrence of Firmicutes and Chloroflexi within the digesters increased with increasing unit methane productivity until the maximum methane productivity was reached. In comparison, Protecbacteria exhibited a declining trend instead (related to C/N balancing and decreasing N content). Aceticlastic *Methanosaetaceae* were the dominant species in the digester, and the hydrogenotrophic methanogens of Methanomicrobiales and Methanobacteria also played a substantial role in biogas production. According to the RSM results, the highest methane production rate that was reached was 8141.32 mL at a 30.06% SS/(CM + MS) ratio, as well as a 15.54 g VS/L feed concentration, which was similar to the observed experimental results. The possible mechanisms could be summarized as follows: (1) the sufficient hydrophilic organics supplied from CW and ultra-pretreated SS; (2) the buffering capacity originating from the CM additive; (3) C/N rebalancing after MS addition and decrease in ammonia toxicity; and (4) the abundant existence of the hydrolyzation bacterial community and archaeal community. Thus, the sufficient hydrophilic component addition, efficient hydrolyzation, and optimal C/N ratio choice guaranteed the high efficiency of the co-digestion.

## Methods

### Chemical characteristics of substrates

SS (feeds) were collected from the secondary sedimentation tank of the Taiping wastewater treatment plant of Harbin (China) and were stored at 4 °C. To enhance the dissolution of the organics within the EPS, the sludge sample was disintegrated by ultrasonication. Specifically, 100 mL of excess sludge was sonicated in a tube using an ultrasonic cell disintegrator using 20 kHz ultrasound, operated at 1.5 W/mL for 10 min. CM was obtained from a dairy farm in Xiangfang District, Harbin. MS was obtained from a suburban farm in Harbin (7–8% moisture content), chopped to a particle size of approximately 1–2 cm, and then pretreated with 6% NaOH solution for 7 days (stirred for 30 minutes every day). Specifically, the feeds of SS, CM and MS had total carbon (TC) contents of 34.5 ± 3.1%, 43.1 ± 1.7% and 30.5 ± 1.1%, with C/N ratios of 5.32, 21.3, and 53.3, respectively. Detailed chemical characteristics of the SS, MS and CM feeds are given in the supplementary materials section.

### Anaerobic digestion operation

Batch digestion tests were carried out in 1-L glass digesters (effective volume of 0.8 L) at a mesophilic temperature of 35 ± 1 °C. Supernatant material from a steady-state operated anaerobic digester was used as inoculum for each digester, which was fully mixed with the feeds before the addition of additives. Each digester was adjusted to a pH of 7.0 ± 0.1 before operation and then flushed with N_2_ for 30 min (300 mL/min) to create an anaerobic environment. The capped digesters were operated for 1 month continuously at 20-d HRT (stirred at 120 r/min); biogas production, TS/VS, SCOD, pH and compositions of VFA were measured every two days.

### Anaerobic co-digestion tests using two different feeds

Batch tests of co-digestion using two feeds, such as SS/CM, CM/MS, and SS/MS, were performed in the abovementioned digesters. First, the abovementioned subtract concentrations were adjusted to 15 g VS/L and then placed in separately operated digesters using a mixing ratio of 3:1 to 2:1 to 1:1 to 1:2 for CM/MS (C/N ratio of 23.99, 25.21, 28.31 and 32.84). Similarly, the feed ratios of SS/MS and SS/CM were adjusted from 2:1 to 1:1 to 1:2 to 1:3, respectively, and the corresponding C/N ratios were 7.46, 9.10, 11.43 and 14.15 for SS/CM co-digestion, and 7.33, 9.19, 12.50 and 17.82 for SS/MS.

### Anaerobic co-digestion tests using three different feeds

Co-digestion using SS, CM and MS as feeds was tested via statistical analysis of a 3 × 3 factorial experimental design (three different feeds ratios × three VS concentrations). The VS concentration was controlled at 10, 15 and 20 g VS/L, respectively, and the different feed ratios were VS_CM_:VS_MS_:VS_SS_ = 40:40:20, VS_CM_:VS_MS_:VS_SS_ = 35:35:30 and VS_CM_:VS_MS_: VS_SS_ = 25:25:50. The performance of the digesters was periodically recorded.

### Extraction and fractionation of organics within sludge and cow manure

Organic material from the experimental SS and CM was extracted following the procedures by Chen *et al*.^28^. In summary, the water content of the sewage sludge (or cow manure) was adjusted to 97.0% first and then centrifuged (4000 × g) for 30 min. Then, 100 g of the solid residue collected after centrifugation was slowly mixed with 200 mL of 25% (v/v) NH_4_OH for 24 h and filtered through a 0.45 *μ*m membrane filter. The collected organics-containing filtrate was diluted (50 times) and acidified to a pH level of 2. The diluted and acidified organics were fractionated into five fractions using XAD-8/XAD-4 resins: a hydrophobic acid (HPO-A), a hydrophobic neutral (HPO-N), a transphilic acid (TPI-A), a transphilic neutral (TPI-N), and a hydrophilic (HPI) fraction. A complete description of the fractionation methods is given in Xue *et al*.^29^.

### DNA extraction, cloning and sequencing

Biomass samples from the digestion reactors were collected and saved for DNA extraction. First, the biomass samples were filtered through 0.22 μm membrane filters (Millipore Laboratories, Billerica, MA) and then stored at −80 °C. DNA in these samples was extracted using UltraClean® Soil DNA Isolation Kits (MoBIO Laboratories, Carlsbad, CA) and subsequently purified via ethanol precipitation. A detailed description of the detection procedure of the archaeal and bacterial community-related DNA can be found in Luton *et al*.^30^ and Fitzgerald *et al*.^31^, respectively.

### Analytical methods

The DOC in the filtrate was analyzed using a TOC-5000 Total Organic Carbon Analyzer (Shimadzu, Kyoto, Japan). The TCOD, SCOD, VS and TS contents of the organics within CM, SS, MS were analyzed according to the Standard Methods^32^. Volatile fatty acids (VFAs) were quantified with a gas chromatograph (Agilent 6890, USA), and the detection limit for VFA analysis was 1.0 mg/L.

## Supplementary information

Supplementary materials-SREP-18-15482C

Dataset 1

## Data Availability

The datasets generated and/or analyzed during the current study are available from the corresponding author on reasonable request (The sequencing data have been deposited in the figshare database (10.6084/84/m9.figshare.750.7502417;17; https://figshare.com/s/58dfaaea91bf6c50d166)).
