# Supplementary material for: Optimization of the co-digestion of sewage sludge, maize straw and cow manure: microbial responses and effect of fractional organic characteristics
Source: Sci Rep. 2019 Feb 20;9:2374. doi: 10.1038/s41598-019-38829-8 (PMC6382933; doi:10.1038/s41598-019-38829-8)
Supplement: Supplementary file 1 — Supplementary materials-SREP-18-15482C [file 41598_2019_38829_MOESM1_ESM.pdf]

Optimization of the co-digestion of sewage sludge, maize straw and cow manure: microbial responses and effect of fractional organic characteristics

Liangliang Wei[1], Kena Qin[1], Jing Ding[1,*], Mao Xue[1], Chaoyong Yang[2], Junqiu Jiang[1] & Qingliang Zhao[1,*]

1. State Key Laboratory of Urban Water Resources and Environment (SKLUWRE); School of Environment, Harbin Institute of Technology, Harbin 150090, China.

2. Tianjin Municipal Engineering Design &Research Institute, Tianjin 300051, China.

* zhql1962@163.com; weill333@163.com (Q.L. Zhao)

Methods

Chemicals characteristics of substrates. SS (feedstock) were collected from the secondary sedimentation tank of Taiping municipal wastewater treatment plant of Harbin (China) and were stored at 4 °C. The sludge had a pH value of 6.9, volatile solids concentration (VS) of 16719~18400 mg/L, total solids concentration (TS) of 26300±1100 mg/L, total chemical oxygen demand (TCOD) of 27400±1800 mg/L and soluble chemical oxygen demand (SCOD) of 816±20 mg/L, respectively. The total carbon (TC) content was 34.5±3.1%, with a C/N ratio of 5.32 (6.5±0.3% for TN). To enhance the dissolution of the organics within the sludge EPS, sludge sample was disintegrated by ultra-sonication. Specifically, 100 mL of excess sludge was sonicated in a tube using an ultrasonic cell disintegrator under 20 kHz ultrasound, and operated at 1.5 W·mL-1 for 10 min.

Cow manure was obtained from a dairy farm in Xiangfang, Harbin, China. The manure had the following characteristics: pH 6.97±0.21; TS of 17.8 ± 1.6% (wet basis); VS of 78.2 ± 4.3% (dry basis); TC of 43.1 ± 1.7% (dry basis), TN of 2.03 ± 0.2% (dry basis); NH4+-N (ammonia nitrogen) of 5321 ± 193 mg/kg (dry basis); total phosphorus (TP) 1.45 ± 0.21% (dry basis), with a C/N ratio of 21.3.

Maize straw was obtained from the suburb farmer of Harbin (7–8% moisture contents), then chopped with a paper chopper to a particle size of approximately 1-2 cm. Those maize straw was pretreated with 6% NaOH solution for 7 days (stirred for 30 minutes every day). The alkaline pretreated maize straw exhibited a TS of 6.3 ± 1.1% (wet basis); VS of 5.1 ± 0.5% (dry basis); TC of 30.5 ± 1.1% (dry basis), TN of 0.57 ± 0.1% (dry basis), with a C/N ratio of 53.3.

DNA extraction, cloning and sequencing. Biomass samples from the digestion reactors under different feedstock ratios were collected and saved for DNA extraction. Firstly, the biomass samples were filtered through 0.22 μm membrane filters (Millipore Laboratories, Billerica, MA) and then stored at -80 oC. DNA in these samples was extracted using UltraClean® Soil DNA Isolation Kits (MoBIO Laboratories, Carlsbad, CA) and subsequently purified via ethanol precipitation. Extracted DNA was quantified using

a NanoDrop spectrophotometer (Thermo Fisher Scientific, Waltham, MA). In order to detect the archaeal community within the digester, gDNA was amplified with primers specific to the mcrA gene according to the PCR protocols (Luton et al., 2002). To enable sample multiplexing during sequencing, barcodes were incorporated between the adapter and forward primer. Triplicate PCRs were performed for each sample, then 454 pyrosequencing was analyzed. Detailed description of the procedural of the bacterial community related DNA could be found in Fitzgerald et al. (2015).

Table S1. Experimental design of Design-Expert 9 and corresponding biogas production under different conditions

Runs	Feed concentration (g VS/L)		Sewage sludge ratio (%)		Biogas production (mL)
	X_1	Code	X_2(SS/(CM+MS))	Code	
1	10	-1	20	-1	6634
2	10	-1	30	0	7425
3	10	-1	50	1	6958
4	15	0	20	-1	7465
5	15	0	30	0	8052
6	15	0	50	1	7235
7	20	1	20	-1	7022
8	20	1	30	0	7658
9	20	1	50	1	7010
